# A Fully Automatic Framework for Parkinson’s Disease Diagnosis by Multi-Modality Images

**DOI:** 10.3389/fnins.2019.00874

**Published:** 2019-08-23

**Authors:** Jiahang Xu, Fangyang Jiao, Yechong Huang, Xinzhe Luo, Qian Xu, Ling Li, Xueling Liu, Chuantao Zuo, Ping Wu, Xiahai Zhuang

**Affiliations:** ^1^School of Data Science, Fudan University, Shanghai, China; ^2^Fudan-Xinzailing Joint Research Center for Big Data, Fudan University, Shanghai, China; ^3^Department of Nuclear Medicine, Daping Hospital, Army Medical University, Chongqing, China; ^4^Department of Nuclear Medicine, North Huashan Hospital, Fudan University, Shanghai, China; ^5^PET Center, Huashan Hospital, Fudan University, Shanghai, China; ^6^Department of Radiology, Huashan Hospital, Fudan University, Shanghai, China

**Keywords:** Parkinson’s disease, multi-modality, image classification, U-Net, striatum

## Abstract

**Background:**

Parkinson’s disease (PD) is a prevalent long-term neurodegenerative disease. Though the criteria of PD diagnosis are relatively well defined, current diagnostic procedures using medical images are labor-intensive and expertise-demanding. Hence, highly integrated automatic diagnostic algorithms are desirable.

**Methods:**

In this work, we propose an end-to-end multi-modality diagnostic framework, including segmentation, registration, feature extraction and machine learning, to analyze the features of striatum for PD diagnosis. Multi-modality images, including T1-weighted MRI and ^11^C-CFT PET, are integrated into the proposed framework. The reliability of this method is validated on a dataset with the paired images from 49 PD subjects and 18 Normal (NL) subjects.

**Results:**

We obtained a promising diagnostic accuracy in the PD/NL classification task. Meanwhile, several comparative experiments were conducted to validate the performance of the proposed framework.

**Conclusion:**

We demonstrated that (1) the automatic segmentation provides accurate results for the diagnostic framework, (2) the method combining multi-modality images generates a better prediction accuracy than the method with single-modality PET images, and (3) the volume of the striatum is proved to be irrelevant to PD diagnosis.

## Introduction

Parkinson’s disease (PD) is the second-most prevalent long-term neurodegenerative disease characterized by bradykinesia, rigidity and rest tremor (Postuma et al., 2015). At present, PD is responsible for about 346,000 deaths per year and is thus one of the major concerns of neuroscience community (Roth et al., 2018). The diagnosis of PD mainly refers to the Movement Disorder Society Clinical Diagnostic Criteria for Parkinson’s disease (MDS-PD Criteria) (Postuma et al., 2015). According to the MDS-PD criteria, the motor symptoms of PD are linked with the loss of dopaminergic neurons, which mainly affects the anatomical regions of the striatum (SARs). Therefore, SARs, which include the caudate nucleus, the putamen and the pallidum, are commonly explored (Strafella et al., 2017).

Functional neuroimaging of the presynaptic dopaminergic system is highlighted according to the MDS-PD criteria (Liu et al., 2018). Positron-emission tomography (PET) is one of the neuroimaging modalities that indicate the regional activity of the tissues. Accordingly, PET tracers are developed to observe the activity of the dopamine transporter (DAT) in early stage of PD, such as ^11^C-CFT, which is a biomarker of the presynaptic dopaminergic system with high sensitivity (Kazumata et al., 1998; Ilgin et al., 1999; Wang et al., 2013). However, due to the low resolution of the PET images, the anatomical and structural information related to the brain that PET can provide is limited. Therefore, the structural neuroimaging methods, such as T1-weighted magnetic resonance imaging (T1-MRI), are introduced to assist the multi-modality diagnosis of PD (Ibarretxe-Bilbao et al., 2011). Bu et al. (2018) worked on the subtypes of multiple system atrophy (MSA) utilizing T1-MRI and ^11^C-CFT PET. Huang et al. (2019) combined these two modalities with ^18^F-FDG PET and analyzed Rapid Eye Movement (REM) Sleep Behavior Disorder research. In both of their studies, T1-MR images were registered to PET images to identify the region of interest (ROIs) in the PET images.

Recently, researchers attempt to improve the accuracy in diagnostic methods with the help of machine learning algorithms, for example, the support vector machine (SVM) has been widely used. Long et al. (2012) used SVM to distinguish early PD patients from NL subjects utilizing resting-state functional MRI, and obtained an accuracy of 86.96%. Haller et al. (2012) used SVM and reached an accuracy of 97% when classifying PD from other atypical forms of Parkinsonism by combining Diffusion Tensor Imaging (DTI) and ^123^I ioflupane Single-Photon Emission Computed Tomography (SPECT). These works combining multi-modality imaging have proved the reliability of artificial intelligence (AI)-assisted PD diagnosis, while few works are reported including ^11^C-CFT PET, to the best of our knowledge.

In this work, we proposed an end-to-end multi-modality diagnostic framework for PD combining T1-MR and ^11^C-CFT PET images. In the framework, MR images were segmented by a U-Net (Ronneberger et al., 2015; Wong et al., 2018). The resulting segmentation was then used to locate the SARs of the PET images by registration. Finally, features extracted from these SARs were used to diagnose PD. Our main contributions include:

(1) We have shown that the automatic segmentation provides accurate results for the proposed diagnostic framework of PD.
(2) We have shown that MR images provide important information to obtain the SAR information in the PET images.
(3) We have demonstrated that the *volume* feature of the striatum is irrelevant to PD diagnosis.

## Methodology

**FIGURE 1:**
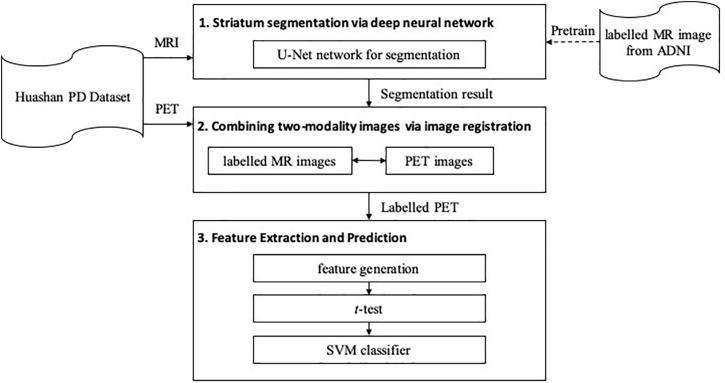
The architecture of the proposed framework.

The proposed framework is shown in Figure 1. It contains three major steps: (1) segmentation, (2) registration, and (3) feature extraction and prediction. In the first two steps, MRI-assisted PET segmentation is performed by MRI segmentation and MRI-PET registration, and in the subsequent step, only information of PET images is considered for PD diagnosis.

### Striatum Segmentation via Deep Neural Network

To obtain the fine structure of the brain tissues, a 3D deep neural network, i.e., U-Net (Ronneberger et al., 2015; Wong et al., 2018), is implemented to segment the striatum in the MR images. The obtained segmentation is used as a reference for SAR localization and extraction in the subsequent procedures.

**FIGURE 2:**
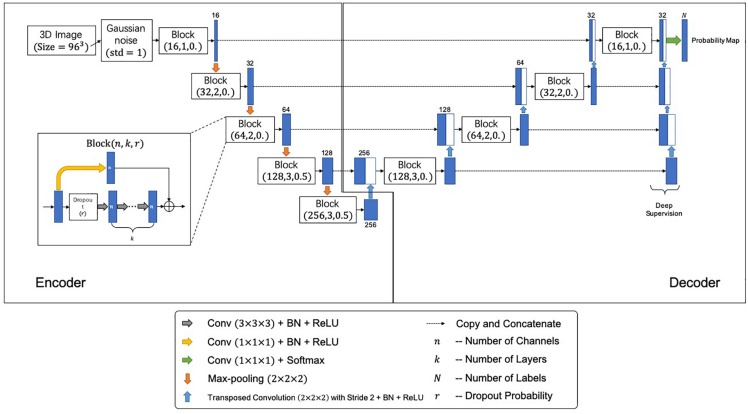
The proposed segmentation network architecture. Each block is represented by (*n*,*k*,*r*), where *n*, *k*, and *r* denote number of channels, number of layers, and the dropout probability, respectively.

Figure 2 shows the network architecture for the segmentation, which outputs a mask indicating the segmented labels of the input image. The network further incorporates the idea of deep supervision introduced by Mehta and Sivaswamy (2017) for faster training convergence. Specifically, the network comprises encoding and decoding paths. The encoding path captures contextual information by residual blocks and max-pooling operations at different resolutions, while the decoding path sequentially recovers the spatial resolution and object boundaries. Besides, skip connections between the upsampled feature maps in the decoder and the corresponding feature maps in the encoder are employed for the combination of local and contextual information. Moreover, the deep supervision scheme is adopted to allow more direct backpropagation to the hidden layers for faster convergence. A final 1 × 1 × 1 convolution layer with a softmax function produces the segmentation probabilities. Gaussian blurring and dropout operations are adopted to avoid overfitting. A loss function is defined to handle the relatively small anatomical structures of labels for accurate segmentation, i.e.,

(1) $$L=w_{D}\,L_{D\,i\,c\,e}+w_{C}\,L_{C\,r\,o\,s\,s},$$

where, *w_D_* and *w_C_* denote the weights of *L_Dice_* and *L_Cross_*, respectively; *L_Dice_* denotes the Dice-related loss, and *L_Cross_* denotes the cross-entropy. They are respectively, given by

(2) $$L_{D\,i\,c\,e}=E_{i}\,\left[{\left(-\ln D\,i\,c\,e_{i}\right)}^{\gamma }\right],$$

with

(3) $$D\,i\,c\,e_{i}=\frac{2\,\left(\sum_{x}\delta_{i\,l}\,\left(x\right)\cdot p_{i}\,\left(x\right)\right)}{\sum_{x}\left(\delta_{i\,l}\,\left(x\right)+p_{i}\,\left(x\right)\right)},$$

and

(4) $$L_{C\,r\,o\,s\,s}=E_{x}\,\left[-\ln p_{l}\,\left(x\right)\right].$$

In Eq. (3), δ*_il_* (*x*) is the Kronecker delta, which equals to 1 if the segmentation label *i* (*x*) equals to the ground-truth label *l* (*x*) at the voxel position *x*, and 0 otherwise; *p_i_* (*x*) is the probability of voxel *x* being labeled as *i*. In our implementations, we chose *w_D_* = 0.8, *w_C_* = 0.2 and γ = 0.3 for the loss function and pretrained the model using an Adam optimizer with a learning rate of 1× 10^−3^ for 10 epochs (Kingma and Ba, 2014). Due to the computational limitations, an ROI of MR images with a size of 96 × 96 × 96 voxels was cropped, which contains the whole structure of SARs.

We employ T1-MR brain images from the Alzheimer’s Disease Neuroimaging Initiative (ADNI^1^) database for pretraining, for the size of the clinical data used in this work is far from being enough for the U-Net training. Note that the information related to AD or other modality data is not used in this study, namely we solely employ the 1859 brain T1-MR images to assist the U-Net training. The ADNI MR images are segmented by the multi-atlas label propagation with the expectation-maximization (MALP-EM^2^) framework (Ledig et al., 2015). The manual segmentation of the caudate nucleus, the putamen and the pallidum are chosen to be the gold standards in the pretraining stage.

### Combining Two-Modality Images via Image Registration

We propose to combine two-modality images for the automatic diagnosis of PD, where T1-MRI provides the morphological information of SARs, and ^11^C-CFT offers pathological information related to PD. The extraction of the SAR information from the MR images is achieved by the DNN segmentation method, as described in Section “Striatum Segmentation via Deep Neural Network.” With this information, one can extract the shape or substructure features from each of anatomical regions. For the combination of the two-modality images, we propose to use image registration, which propagates the anatomical and structural information of SARs in the MRI to the PET.

The registration in the multi-modality diagnostic framework is achieved via the zxhproj^3^ platform (Zhuang et al., 2011). Firstly, the image with prior label information is registered to the target PET image. The resulting transformation is then used to propagate the prior label information to the PET, which results in the automatic localization of the SARs for the target PET image. Since the MRI and PET images are from the same subject at the same acquisition session, we propose to use a rigid registration. By registration, the caudate nucleus, the putamen and the pallidum, as well as the parieto-occipital regions are labeled.

For comparisons, we propose a single-modality diagnostic framework using solely PET images. To achieve the fully automated diagnosis, we propose to achieve the anatomical information in the PET images via the same registration method used for the multi-modality scheme. In this scenario, the image with prior label information is defined using a pre-labeled PET template, and the registration between the template and the target PET is achieve via an affine registration following a pre-rigid registration.

### Feature Extraction and Prediction

To extract adequate features from the SARs, the caudate nucleus and the putamen are further divided into three substructures using a k-means algorithm (Ng et al., 2006). After clustering, statistics of image intensity are calculated to represent the feature information in each region, including *maximum*, *minimum*, *median*, *1st* and *3rd quantile*, and *mean* of PET intensity. Several studies characterize radioactive uptake by the striatal-to-occipital ratio (SOR), as the parieto-occipital region is widely considered of lacking CFT uptake (Ma et al., 2002; Carbon et al., 2004; Huang et al., 2007). In this work, the SOR, which is defined as *(striatum-occipital)/occipital*, is calculated with each kind of intensity value. Meanwhile, the *volumes* of the six anatomical SARs are included into the feature set. In all, 90 features are generated (for a list of specific features, see Supplementary Table S1).

After feature extraction, a *t*-test is performed to analyze the significance of each feature. Setting significance level α = 0.01, features with a *p*-value less than 0.01 are considered as being statistically significant. Only significant features would be regarded as the arguments of the machine learning models.

Consequently, the SVM classifier is trained to classify the subjects (Haller et al., 2012; Long et al., 2012). Furthermore, to estimate the generalization ability and stability of the method, the leave-*n*-out cross-validation strategy is employed to evaluate the performance of the models. In addition, we implement the random forest algorithm to calculate the importance of the features (Gray et al., 2013).

## Experiments

The following parts in this section are organized as follows. Section “Data Acquisition” describes the data used in this work; Section “Evaluation of Automatic Striatum Segmentation” validates the reliability of the automatic segmentation method; Section “Advantages of Multi-Modality Images” investigates the advantages of combining multi-modality images; and Section “Efficacy of Volume Features” explores the efficacy of the volume features of SARs for the diagnostic of PD.

### Data Acquisition

Data used in this study was collected from the Department of Neurology, Huashan Hospital, Fudan University. It contains paired ^11^C-CFT PET and T1-MR images of PD patients and healthy participants. MR images were acquired by a 3.0-T MR scanner (DiscoveryTM MR750, GE Healthcare, Milwaukee, WI, United States). Each MR image was visually inspected to rule out motion artifacts (Bu et al., 2018; Huang et al., 2019). PET images were acquired by a Siemens Biograph 64 PET/CT scanner (Siemens, Munich, Germany) in three-dimensional (3D) mode. A CT transmission scan was first performed for attenuation correction. Static emission data were acquired 60 min after the intravenous injection of 370 MBq of ^11^C-CFT and lasted for 15 min. All subjects were scanned in a supine position with a dimly lighted and noise-free surrounding (Bu et al., 2018; Huang et al., 2019). The synchronous MRI data were acquired using a T1-weighted 3D inversion recovery spoiled gradient recalled acquisition (IR-SPGR) with the following parameters: TE/TR = 2.8/6.6 ms, inversion time = 400 ms, flip angle = 15°, matrix = 256 × 256 × 170, field-of-view = 24 cm, and slice thickness = 1 mm. MR and PET images acquisition for each subject had a time interval of no more than 3 months.

Forty-nine patients with PD and 18 age-matched normal control (NL) subjects were recruited. All subjects were screened and clinically examined by a senior investigator of movement disorders before entering the study and were followed up for at least 1 year. The diagnosis of PD was made referring to the MDS-PD Criteria. The Unified Parkinson’s Disease Rating Scale (UPDRS) and Hoehn and Yahr scale (HY) were assessed after the cessation of oral anti-parkinsonian medications (if used) for at least 12 h. The following exclusion criteria were used for the NL subjects’ recruitment: (1) being tested positive by the REM Sleep Behavior Disorder Single-Question Screen (Postuma et al., 2012), (2) a history of neurological or psychiatric illness, (3) a prior exposure to neuroleptic agents or drugs, (4) an abnormal neurological examination. The data are summarized in Table 1. In this study, gender proportion differences between groups could be ignored, as previous studies have shown no significant difference in DAT bindings between genders (Eshuis et al., 2009). The research was approved by the Ethics Committee of Huashan Hospital. All subjects or legally responsible relatives signed written informed consent in accordance with the Declaration of Helsinki before the study.

**TABLE 1:** Summary for the studied dataset.

**Subject**	**HY**	**Count**	**Gender (M/F)**	**Age**	**UPDRS**
NL	0	18	4/14	64.1 ± 6.7	–
PD	1	15	10/5	61.2 ± 7.6	14.3 ± 5.1
	2	26	16/10	62.0 ± 7.9	21.6 ± 7.5
	3	8	4/4	58.8 ± 5.9	34.6 ± 7.4

*For gender, the expression means Male/Female, and for age and Unified Parkinson’s Disease Rating Scale, the expression means mean ± standard deviation.*

After data acquisition, both sides of the caudate nucleus, the putamen and the pallidum of each MR image were manually labeled by an experienced clinician from the Department of Neurology, Huashan Hospital. To ensure the qualities of the segmentation results, boundaries of these anatomical structures were double-checked by another clinician from the same department.

### Evaluation of Automatic Striatum Segmentation

**FIGURE 3:**
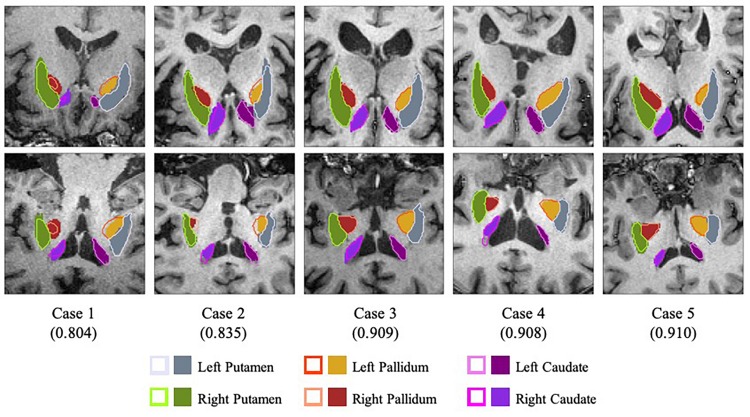
Visualization of the segmentation results with slices of the axial view (top row) and the coronal view (bottom row). Case 1 and case 2 are two worst segmentation results, and case 3, case 4 and case 5 are three median results. Values in the parentheses refer to the corresponding dice similarity coefficients. As for the legends, the colored contours represent the automatic segmentation boundaries while the colored blocks are the corresponding ground truth masks.

To test the performance of the segmentation network, three-fold cross-validation was performed. The whole dataset was split into three disjoint parts, and the model was fine-tuned for 5 epochs on the union of every two disjoint subsets. Table 2 illustrates the average Dice Similarity Coefficient (DSC) of each anatomical region, and Figure 3 provides a visualization of the segmentation results of five example cases. One can find that the left pallidum (colored goldenrod in Figure 3) is worst segmented with the maximal standard deviation while the right putamen (colored olive drab in Figure 3) is best segmented with the minimal standard deviation.

**TABLE 2:** Average DSCs of the segmentation of each anatomy.

	**Right caudate**	**Left caudate**	**Right pallidum**	**Left pallidum**	**Right putamen**	**Left putamen**
DSCs	88.5 ± 6.3%	90.1 ± 7.2%	89.3 ± 11.4%	86.9 ± 13.0%	92.2 ± 5.0%	91.4 ± 5.5%

**FIGURE 4:**
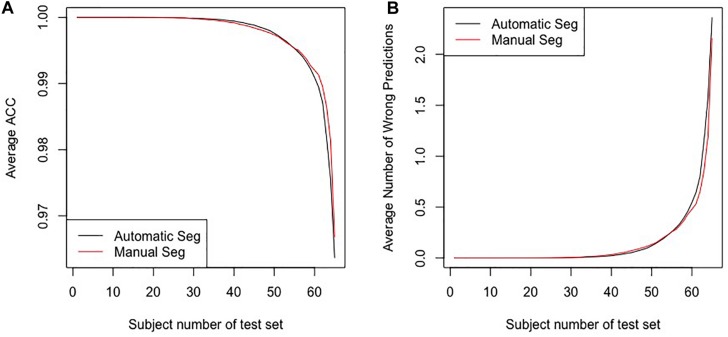
The results for the leave-*n*-out cross-validation of the classification with automatic segmentation and manual segmentation. Panel **(A)** presents the average ACC, and panel **(B)** presents the average number of wrong predictions. The horizontal axes in the two panels represent subjects numbers of the test set, i.e., the *n* in the leave*-n*-out cross-validation.

Figure 4 shows the average accuracy (ACC) and the number of wrong predictions with leave-*n*-out cross-validation of the different segmentation methods, i.e., automatically and manually. Both accuracies reached 100% when *n* = 1, and the accuracies and the numbers of wrong predictions of the two experiments result in no significant difference in a pairwise *t*-test (*p*-value = 0.1017). Furthermore, when training classifiers using features of manually segmented images and testing it using features of automatically segmented images, we still obtained 100% accuracy. All results indicate that the automatic segmentation provides accurate results for the proposed diagnostic framework of PD.

### Advantages of Multi-Modality Images

To evaluate the influence of multi-modality images, the single-modality method using solely PET images was compared. In the multi-modality scheme, the MR images provides accurate anatomical and structural SAR information of the subject. By contrast, in the single-modality method this information is achieved by registering the PET images to a pre-labeled Automated Anatomical Labeling (AAL) PET template. We conducted the rest of the pipeline in the same way for the two methods.

**FIGURE 5:**
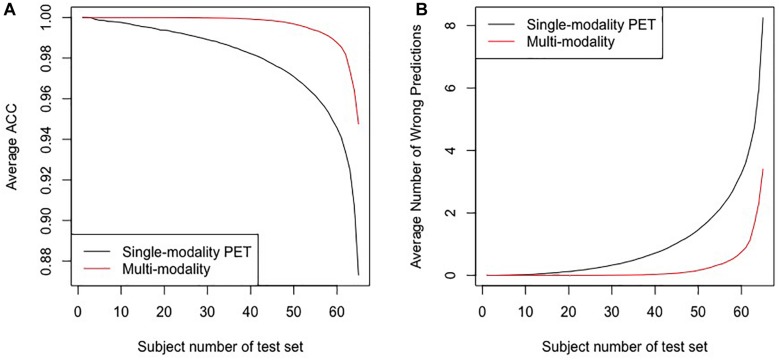
The results for the leave-*n*-out cross-validation of the classification by the multi-modality diagnostic method and the single-modality method. Panel **(A)** presents the average ACC, and panel **(B)** presents the average number of wrong predictions. The horizontal axes in the two panels represent subjects numbers of the test set, i.e., the *n* in the leave*-n*-out cross-validation.

Figure 5 shows the results of the comparative experiments with leave-*n*-out cross-validation. The results demonstrate that with the assistance of MR images, the performance of the multi-modality group is better than the single-modality PET group in the PD/NL task. When *n* = 1, the accuracy of the multi-modality group reached 100% in the PD/NL task, while the accuracy of the single-modality PET group was 98.51%.

To test the uniformity of the classifiers based on the different groups, we also trained the classifier using features of multi-modality images and tested it using features of single-modality PET, and the accuracy dropped to 88.05%, with 8 subjects misclassified.

### Efficacy of Volume Features

In the feature extraction step, *t*-tests were performed to evaluate the significances of all feature, and results indicated that the features of the *volume* are not statistically significant with α = 0.01 (see Supplementary Table S1 for more details). To further evaluate the effects of the *volume* of SARs, we compared the importance of different features based on groups with and without *volume*, as Table 3 shows. One can see that the importance values of the two groups are similar. Hence, the effect of *volume* to the model is negligible. Note that the *volume* is calculated based on the original MR images without downsampling.

**TABLE 3:** Feature importance of groups with/without *volume*.

**Feature**	**Group with *Volume***	**Group without *Volume***
*mean*	0.2030	0.2006
*median*	0.2024	0.1965
*3rd quantile*	0.1961	0.1946
*1st quantile*	0.1825	0.1835
*maximum*	0.1443	0.1497
*minimum*	0.0715	0.0751
*volume*	0.0002	–

*The importance values were calculated by the random forest algorithm.*

## Discussion and Conclusion

In this work, we proposed a fully automatic framework for PD diagnosis. This method utilized two modalities, i.e., ^11^C-CFT PET and T1-MR imaging, performed MRI-assisted PET segmentation, selected features and employed SVM to give the predictions. To validate the performance of the framework, we applied the proposed method on the clinical data from Huashan Hospital.

One of the major differences between the proposed method and the traditional methods is that the SARs are located according to the labels of the automatic segmentation by U-Net. To evaluate the performance of the U-Net, we calculated the DSCs between automatic and manual segmentation. In addition, we compared the proposed pipeline, whose SARs were located according to the automatic segmentation, to the method whose SARs were manually segmented. The leave-*n*-out experiment shows the two methods performed comparably, indicating that the automatic segmentation could provide accurate results for the proposed diagnostic framework of PD. Further investigation of the feature importance of the two groups is illustrated in Table 4. It indicates that the *minimum* has lower importance than the first five features. Given that the striatum region has a higher uptake value compared with its adjacent areas, voxels with minimal intensity value are more likely to appear on the edge of the SARs. Therefore, the inaccurate delineation of the anatomical boundary as a potential result of the automatic segmentation could not cause a significant decline in the performance of the overall diagnostic framework.

**TABLE 4:** Feature importance of groups with manual segmentation results and automatic segmentation results.

**Feature**	**Manual segmentation**	**Automatic segmentation**
*mean*	0.2064	0.2031
*median*	0.2010	0.2025
*3rd quantile*	0.2008	0.1962
*1st quantile*	0.1654	0.1825
*maximum*	0.1660	0.1443
*minimum*	0.0605	0.0716

*The importance values were calculated by the random forest algorithm.*

**FIGURE 6:**
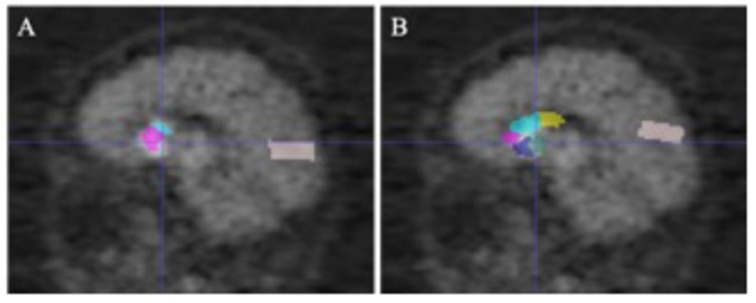
The comparison of gold standard and wrongly placed SARs of the wrongly predicted subject. Panel **(A)** shows the segmentation result in gold standard, and panel **(B)** shows the segmentation in the wrongly predicted subject. Images are in sagittal plane and have the same cursor position.

An alternative way to locate SARs for subsequent feature extraction is to apply a pre-labeled PET template by registration. In Section “Advantages of Multi-Modality Images,” AAL PET template was used as the PET template, and was registered to PET images for the localization of SARs. Experiments show that the diagnostic capability of this single-modality PET group is worse than the proposed multi-modality framework. Though the single-modality PET approach gives a favorable prediction, the multi-modality approach performs better. This is because the localization of the SARs occupies a significant place in the diagnostic framework, and the additional structural information from MR images can better locate SARs. Figure 6 demonstrates that the single-modality PET approach might be affected by the erroneous delineation of the SARs. The error could be attributed to the ignored inter-subject variations in brain structures when defining SARs from a PET template.

To test the uniformity of the classifiers based on different segmentation approaches, we trained classifiers using features of manually segmented multi-modality images, and tested it using features of other methods. When testing with features of multi-modality automatic segmentation method, we still obtained 100% accuracy, indicating that the features of manual and automatic segmentation are highly consistent. However, testing with the single-modality method resulted in an accuracy of 88.05%. The lower accuracy might be explained by the lack of adequate extracted features due to the falsely located SARs. Hence, compared with the multi-modality group, single-modality PET group naturally needs more feature engineering and better-designed algorithms.

**FIGURE 7:**
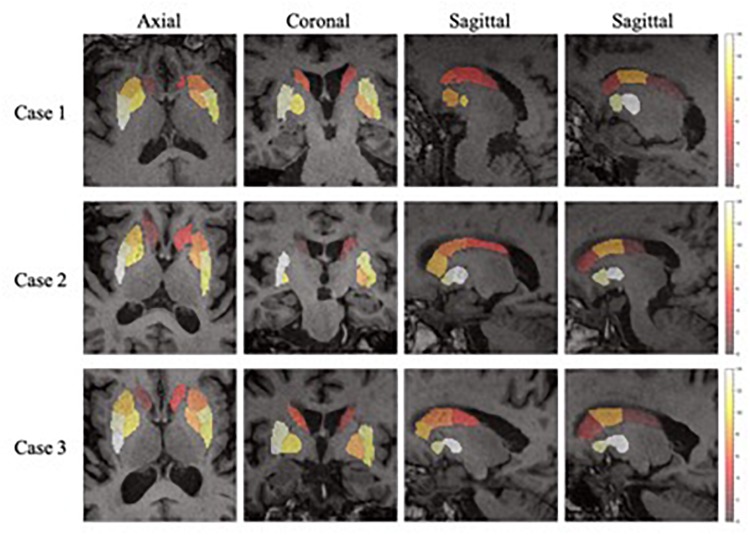
The importance of the SARs in the proposed framework. One axial slice, one coronal slice and two sagittal slices (right and left side of the regions, respectively) of three subjects are chosen to show the importance of the SARs.

In the feature extraction, features of *volume* were rejected according to the *t*-test. This could be the reason why the volume of SARs does not change significantly with the progression of PD, as concluded from the literature (Ibarretxe-Bilbao et al., 2011). Figure 7 shows the heatmaps of feature distribution on the SARs, displaying the influence of each subregion for the classification in the PD/NL task. The difference of influence is expressed by the color scale. One can find that the most relevant region influencing the separation of PD/NL is localized in the middle and rear of the putamen, then the pallidum, and the caudate nucleus reveals the least significance on this task.

Several future studies could be completed explored based on the current pipeline. Firstly, the classifiers can be trained with Parkinsonian disorders (PDS) dataset to classify PD and atypical PDS, such as MSA and Progressive Supranuclear Palsy (PSP), which has important clinical values but is with great challenges. Secondly, this framework only contains medical imaging information currently, while other aspects of information, such as age, gender, motor ratings and other biomarkers are not included, which may further improve the diagnostic accuracy. Future research could be undertaken to incorporate additional multimodal data for better disease prediction. Finally, the sample size of subjects in this work is relatively small, and a bigger dataset is expected to validate our experiment results and improve the performance of the framework.

To conclude, we proposed a fully automatic framework combining the two modalities for PD diagnosis. This framework obtained a promising diagnostic accuracy in the PD/NL task. In addition, this work also emphasized the high value of the ^11^C-CFT PET in the PD diagnosis.

## Data Availability

The datasets generated for this study are available on request to the corresponding author.

## Ethics Statement

All data were collected from Department of Neurology, Huashan Hospital, Fudan University, and the study was approved by the Ethics Committee of Huashan Hospital. All subjects or a legally responsible relative were given written informed consent before the study.

## Author Contributions

XZ is the principle investigator of this work, designed the experiments, and supervised and revised the manuscript. PW co-investigated the research and revised the manuscript. CZ co-investigated the research. JX led the implementation and experiments and wrote the manuscript. YH co-led the work and wrote the manuscript. XiL provided support to the work of coding, experiments, and manuscript writing. FJ, QX, and LL collected the data. XuL collected the data and segmented the striatum of the subjects.

## Conflict of Interest Statement

The authors declare that the research was conducted in the absence of any commercial or financial relationships that could be construed as a potential conflict of interest.

## Footnotes

1 adni.loni.usc.edu

2 https://biomedia.doc.ic.ac.uk/software/malp-em/

3 http://www.sdspeople.fudan.edu.cn/zhuangxiahai/0/zxhproj/
