# Supplementary material for: A Fully Automatic Framework for Parkinson’s Disease Diagnosis by Multi-Modality Images
Source: Front Neurosci. 2019 Aug 23;13:874. doi: 10.3389/fnins.2019.00874 (PMC6716425; doi:10.3389/fnins.2019.00874)
Supplement: Supplementary file 1 [file Data_Sheet_1.docx]

**Table 1** *p*-values of *t*-test of the features in the proposed framework. Setting significance level $\alpha=0.05$, features with a *p*-value less than 0.05 are considered as significance features.

| Regions | | SOR | | | | | | volume |
| --- | --- | --- | --- | --- | --- | --- | --- | --- |
|  |  | *mean* | *max* | *min* | *1^st^ quantile* | *median* | *3^rd^ quantile* |  |
| Right Caudate | front | 6.52E-10 | 2.55E-09 | 4.65E-06 | 2.87E-09 | 1.84E-09 | 3.41E-10 | 0.0544 |
|  | middle | 7.02E-10 | 4.76E-10 | 0.001585 | 6.32E-09 | 7.02E-10 | 2.46E-10 |  |
|  | rear | 1.04E-07 | 1.78E-09 | 2.21E-07 | 3.94E-06 | 6.00E-07 | 3.06E-08 |  |
| Left Caudate | front | 1.11E-08 | 2.70E-08 | 3.20E-07 | 2.75E-08 | 2.11E-08 | 8.46E-09 | 0.0321 |
|  | middle | 5.52E-08 | 4.72E-08 | 2.51E-05 | 1.65E-07 | 6.76E-08 | 4.74E-08 |  |
|  | rear | 1.41E-06 | 2.03E-07 | 0.000103 | 3.78E-06 | 3.45E-06 | 1.82E-06 |  |
| Right Putamen | front | 7.37E-18 | 2.80E-16 | 3.16E-07 | 2.03E-17 | 1.64E-17 | 1.19E-17 | 0.0469 |
|  | middle | 1.18E-30 | 7.62E-26 | 1.13E-09 | 5.21E-14 | 6.58E-31 | 1.79E-30 |  |
|  | rear | 1.03E-13 | 1.57E-13 | 1.02E-07 | 1.20E-12 | 1.50E-13 | 6.86E-14 |  |
| Left Putamen | front | 9.50E-15 | 5.80E-15 | 2.11E-06 | 5.24E-14 | 3.66E-14 | 8.96E-15 | 0.02 |
|  | middle | 1.01E-27 | 3.03E-22 | 2.17E-10 | 1.80E-28 | 2.36E-28 | 5.97E-27 |  |
|  | rear | 3.26E-31 | 8.34E-14 | 9.98E-11 | 3.18E-29 | 1.02E-30 | 9.95E-32 |  |
| Right Pallidum | | 8.84E-23 | 4.07E-21 | 7.53E-07 | 4.72E-16 | 1.27E-21 | 1.60E-12 | 0.002 |
| Left Pallidum | | 6.06E-19 | 1.50E-17 | 4.87E-07 | 1.75E-07 | 2.90E-08 | 2.13E-09 | 0.309 |
